# Uncoupling protein-1 deficiency promotes brown adipose tissue inflammation and ER stress

**DOI:** 10.1371/journal.pone.0205726

**Published:** 2018-11-14

**Authors:** Laura M. Bond, Maggie S. Burhans, James M. Ntambi

**Affiliations:** 1 Department of Biochemistry, University of Wisconsin–Madison, Madison, Wisconsin, United States of America; 2 Department of Nutritional Sciences, University of Wisconsin–Madison, Madison, Wisconsin, United States of America; State University of Rio de Janeiro, BRAZIL

## Abstract

Inflammation and endoplasmic reticulum (ER) stress are hallmarks of metabolic syndrome. While these metabolic derangements have been well-investigated in white adipose tissue, their existence and etiology in brown adipose tissue (BAT) are poorly understood. Here, we aimed to investigate ER homeostasis and the inflammatory status and of BAT lacking uncoupling protein-1 (UCP1), a protein required for BAT thermogenesis. H&E staining illustrated lipid accumulation and crown-like structures surrounding adipocytes in BAT of UCP1-/- mice housed at room temperature compared to control mice. Further, immunohistological evaluation of F4/80 and gene expression studies demonstrated BAT macrophage infiltration and robust elevation of pro-inflammatory markers in UCP1-/- BAT. ER stress was also present in BAT of UCP1-/- mice, as evidenced by elevated gene expression and post-translational modifications of unfolded protein response components. After four weeks of thermoneutral housing, UCP1-/- mice did not exhibit elevated BAT inflammation and ER stress gene expression compared to WT mice, but depot expansion persisted. Collectively, we demonstrate that the effects of UCP1 deficiency in BAT are not restricted to mitochondrial uncoupling. We conclude that brown adipose tissue of UCP1-/- mice exhibits pro-inflammatory immune cell infiltration and perturbations in ER homeostasis and that this phenotype is driven by cold exposure rather than lipid accumulation.

## Introduction

Inflammation and endoplasmic reticulum (ER) stress are implicated in numerous metabolic disorders, including obesity, Type I diabetes, and Type II diabetes [1, 2]. WAT adipose tissue in obesity is characterized by chronic, low-grade inflammation, and the immune cell population is estimated to shift from 10% to 50% during obesification of WAT of mice [3] as pro-inflammatory macrophages and immune cells infiltrate the depot [2]. ER stress activates the unfolded protein response (UPR), a tripartite molecular system that aims to suspend broader protein synthesis and promote protein folding via protein chaperones. These two pathologies are intertwined and serve a potent feedforward mechanism, both contributing to metabolic disease [1]. Cytokines secreted by pro-inflammatory macrophages in WAT directly contribute to insulin resistance [4], and the ER senses metabolic stress and can disrupt glucose homeostasis [5, 6]. While WAT inflammation and ER stress have received significant investigative attention, fewer instances of inflammation and ER stress have been reported in BAT [7–10].

Uncoupling protein-1 (UCP1) is a mitochondria-resident protein that mediates heat production in brown adipose tissue by uncoupling the electron transport chain from ATP synthesis. At room temperature [11], UCP1-/- mice are under chronic cold stress at room temperature, but compensatory mechanisms of thermogenesis enable animals to maintain core body temperature [12–14]. UCP1-/- mice are widely used for investigating BAT thermogenesis, yet we have an incomplete understanding of the BAT phenotype of this mouse model. Specifically, the interplay between UCP1 and the BAT stress response is not well-characterized. We hypothesized that loss of UCP1 would yield lipid accumulation and brown adipocyte hypertrophy. Further, because lipid accumulation has been linked with inflammation and ER stress in WAT [2, 3, 5], we speculated that BAT expansion in UCP1-/- mice would be accompanied by immune cell infiltration and perturbations to ER homeostasis. Indeed, recently published proteomic and microarray data suggest an immune response in BAT of UCP1-/- mice [7, 15]. Additionally, UCP1-/- mice exhibit impaired mitochondrial calcium buffering capacity and ROS [7, 16], though the effects of UCP1 deficiency on oxidative stress and mitochondrial dysfunction are debated [17, 18]. These reports warrant an investigation into ER homeostasis in BAT of UCP1-/- mice and a hypothesis-driven characterization of the inflammatory response. As such, we sought to determine if UCP1 depletion elicits pro-inflammation and activation of the unfolded protein response.

We previously reported a splicing mutation in UCP1 that causes loss of UCP1 protein [19]. Using this mouse model in the present study, we demonstrate that loss of UCP1 elicits dramatic deleterious changes in ER homeostasis and inflammation. BAT from UCP1-/- mice showed evidence for perturbations in brown adipose tissue ER homeostasis as well as macrophage infiltration and pro-inflammation at room temperature. Additionally, we housed WT and UCP1-/- mice at thermoneutrality and determined that elimination of chronic cold stress largely rescues this stress response but, surprisingly, not brown fat depot expansion.

## Materials and methods

### Animals

UCP1-/- mice were generated as described [19]. Briefly, stearoyl-CoA desaturase-3 flox (SCD3F/F) mice were created by cloning a construct in which *Scd3* exon 3 was flanked by LoxP sites. SCD3F/F mice were crossed with eIIa cre+/- mice (Jackson Laboratory) to globally delete SCD3. After germline deletion was achieved, cre was bred out of SCD3-/- mice. The *Ucp1* mutation was discovered after generation of SCD3-/- mice and was not present in the SCD3F/F line. *Ucp1* SNP and exon 5 deletion were demonstrated in SCD3-/- mice using Sanger sequencing [19]. After UCP1 mutation discovery, the UCP1 and SCD3 mutations were bred apart and line to generate SCD3F/F;UCP1-/- mice, which have two functional copies of SCD3 and are herein called UCP1-/- mice. Mice were genotyped for the *Ucp1* SNP using UCP1 exon 5 PCR amplification followed by MspI restriction digest. All studies were conducted with UCP1-/- mice compared to littermate UCP1+/+ controls, and only male mice were used in this study. Canonical UCP1-deficient mice (purchased from Jackson Laboratory, called UCP1(J)-/- mice) and littermate UCP1+/+ mice were used as indicated. As described previously, the UCP1-/- mice used by our group are phenotypically equivalent to canonical UCP1(J)-/- mice; they exhibit modest cold sensitivity and the same body and tissue weight trends [19].

Mice were maintained on a 12-hour light-dark cycle (6AM to 6PM) and had free access to food and water until the day of sacrifice. All animals were bred, born and weaned at room temperature. Mice were fed a standard chow diet (Purina 5008) after weaning and until sacrifice. At 11–12 weeks of age, mice were fasted 4 h and euthanized by isoflurane overdose beginning at 10AM. Blood was collected via cardiac bleed and tissues were collected and frozen. For room temperature experiments, mice were housed at 21°C in the animal care facility at the University of Wisconsin–Madison department of biochemistry. Thermoneutrality housing experiments (30°C) were four weeks in length and were completed at the University of Wisconsin–Madison Biotron. All in vivo experimental animal procedures were approved by the Institutional Animal Care and Use Committee of the University of Wisconsin–Madison (protocol A005125). Animals were euthanized by isoflurane overdose followed by cardiac puncture to ensure death.

### Real-time quantitative PCR

Total RNA was isolated using RNeasy Lipid Tissue Mini Kit (Qiagen). cDNA was synthesized from RNA using High Capacity cDNA Reverse Transcription Kit (Applied Biosystems). Quantitative reverse-transcriptase PCR (qPCR) was performed using Power SYBR Green Master Mix and ABI7500 instrument. Relative mRNA abundance was calculated as relative Ct value and normalized to housekeeping gene, *Arbp* or *18S*. Primer sequences may be found in S1 Table.

### Immunoblotting

Adipose tissue homogenates were prepared using RIPA buffer (Cell Signaling Technology) supplemented with 1 mM PMSF and phosphatase inhibitor cocktail (0.5 mM imidazole, 0.25 mM sodium fluoride, 0.3 mM sodium molybdate, 0.25 mM sodium orthovanadate, and 1.0 mM sodium tartrate). Protein concentrations were measured using a modified Lowry assay. 25–40μg protein were separated by SDS-PAGE gel (10% acrylamide) and transferred onto PVDF membrane. Membranes were blocked in 5% non-fat dry milk (w/v) for one hour then treated with primary antibody overnight at 4°C. Antibodies from Cell Signaling Technology include: eIF2α (#5324, 1:1000), phosphor-Ser51 eIF2α (#3398, 1:1000), vinculin (#4650, 1:1000), tubulin (#3873, 1:3000). Antibodies from Abcam include: OXPHOS cocktail (ab110412, 1:1000), UCP1 (ab10983, 1:2000). Antibodies from Millipore include: GAPDH (MAB374, 1:2000).

### Histology and immunohistochemistry

Fresh BAT was fixed in formalin then paraffin-embedded for sectioning and staining with hematoxylin and eosin. For F4/80 staining, BAT was frozen in OCT prior to sectioning and F4/80 staining. Reagents include: F4/80 primary antibody (1:400 AbdSerotec, MCA497G), anti-rat IgG (Vector, MP-7444), DAB substrate (Cell Signaling Technology), Mayer’s hematoxylin (Sigma). Images were acquired using a Nikon Eclipse Ti Intensilight microscope at 40x magnification.

### Statistics

Statistical analyses were performed using student’s *t*-test (2-sided, unpaired). All data are reported as mean ± SEM. Statistical significance of WT to UCP1-/- mice is denoted by *p<0.05.

## Results

### UCP1-/- mice exhibit BAT macrophage infiltration and elevated pro-inflammatory cytokine production

**Fig 1:**
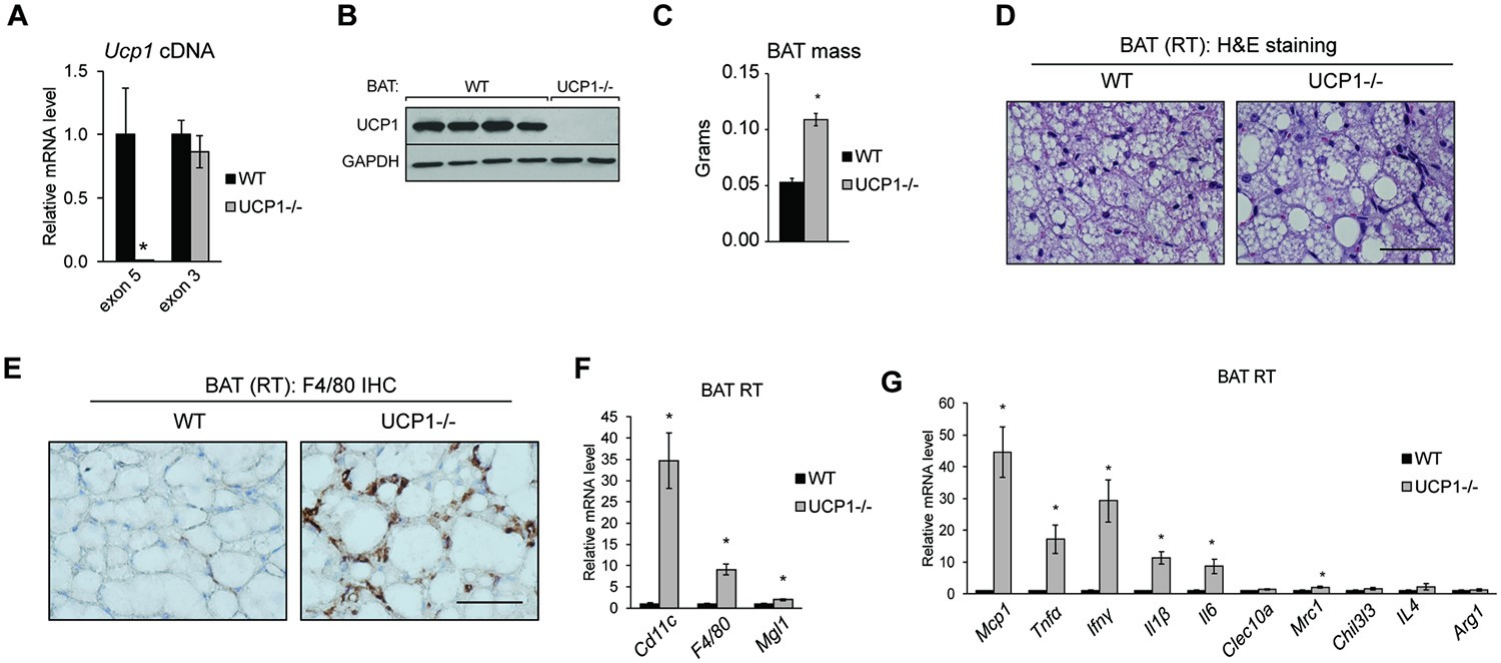
UCP1 deficiency promotes BAT expansion and inflammation. Chow-fed, male mice were housed at room temperature (21°C) and sacrificed at 12 weeks of age (n = 5-7/group). (A) *Ucp1* exon 5 and exon 3 transcript abundance as measured by qRT-PCR. (B) Immunoblots of UCP1 in WT and UCP1-/- BAT. BAT mass (C) and H&E staining (D) of WT and UCP1-/- mice. Scale bar represents 50μm. (E) F4/80 immunohistochemistry of brown adipose tissue from WT and UCP1-/- mice. BAT was cryosectioned and stained with F4/80 and DAB. Scale bar represents 50μm. (F) qRT-PCR analysis of gene markers of macrophage (F) and cytokine and chemokine (G) content. Data are mean ± SEM. *p<0.05 vs WT.

We previously reported a splicing mutation in *Ucp1* that causes loss of UCP1 [19]. These mice have a deletion in *Ucp1* exon 5 and lack UCP1 protein (Fig 1A and 1B). Here, we probe the effects of UCP1 deficiency on the BAT health. Mice lacking UCP1 exhibited brown adipose tissue expansion compared to wildtype littermate controls (Fig 1C), and histological evaluation illustrated that UCP1-/- brown adipocytes contained larger lipid droplets compared to WT brown adipocytes (Fig 1D). In addition to increased LD size, H&E staining revealed crown-like structures around UCP1-/- brown adipocytes, and we used F4/80 immunohistochemistry to confirm that the cells encapsulating UCP1-/- adipocytes were macrophages (Fig 1E). *F4/80* gene expression was elevated 9-fold in whole BAT (Fig 1F). We next investigated the nature of the inflammatory response in BAT. We observed a robust upregulation of pro-inflammatory markers in BAT of UCP1-/- mice housed at room temperature (21°C) (Fig 1G). Specifically, *Mcp1*, monocyte chemoattractant protein-1, was elevated 40-fold and *Cd11c* was elevated 35-fold. Consistent with a pro-inflammatory state, we observed a strong increase in transcript levels of the cytokines, *Ifnγ*, *Tnfα*, *Il-1β* and *Il-6*. Expression of *Mgl1*, a marker of anti-inflammatory macrophages, was modestly increases, but expression of anti-inflammatory markers (*Clecl0a*, *Mrc1*, *Chil3l3*, *Il4*, and *Arg1*) were minimally altered in UCP1-/- mice compared with WT controls (Fig 1G).

### UCP1 deficiency promotes ER stress and mitochondrial dysfunction

**Fig 2:**
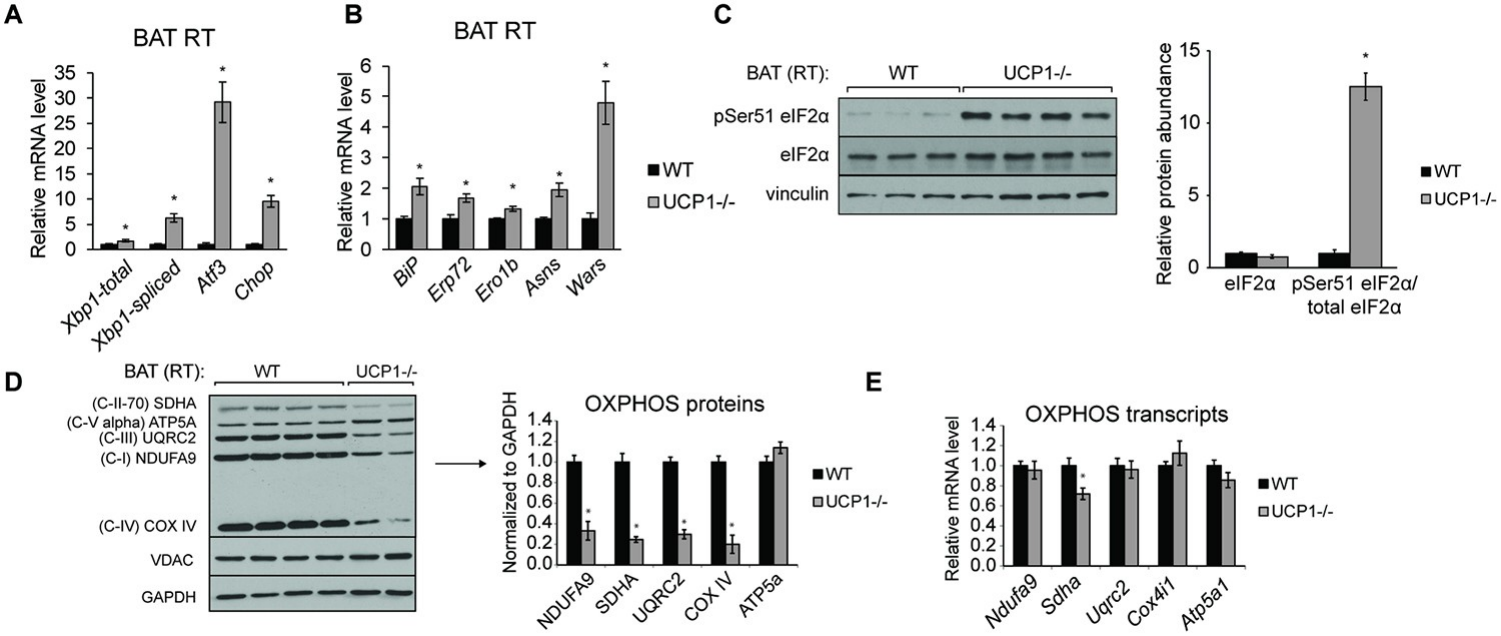
Loss of UCP1 activates the ER stress response in BAT. Chow-fed, male mice were housed at room temperature (21°C) and sacrificed at 12 weeks of age (n = 5-7/group). qRT-PCR analysis of transcription factors (A) and chaperones and amino acid metabolism (B) genes associated with the unfolded protein response (n = 5-7/group). (C) Immunoblotting of eIF2α phosphorylation at Ser51 in WT and UCP1-/- brown adipose tissue. OXPHOS protein (D) and transcript (E) levels of BAT from WT and UCP1-/- mice housed at room temperature. Data are mean ± SEM. *p<0.05 vs WT.

We next investigated markers of ER stress, since ER stress is a common corollary of inflammation [1]. We detected 10-fold and 35-fold increases in *Chop* and *Atf3* transcript levels, respectively (Fig 2A). Abundance of the spliced Xbp1 transcript was also significantly increased (Fig 2A), indicating activation of the IRE1 branch of the UPR. Transcript levels of chaperones and amino acid synthesis genes were modestly but significantly increased (Fig 2B). Phosphorylation of eIF2α, a PERK substrate and mediator of translation repression, was robustly elevated in UCP1-/- BAT compared to WT BAT (Fig 2C).

Because UCP1 is located adjacent to the respirasome in the inner mitochondrial membrane and mitochondrial function is often compromised in models of inflammation and ER stress [20], we asked how UCP1 deficiency alters mitochondrial OXPHOS function. We saw that UCP1-deficient BAT had reduced protein levels of complex I-IV, comprising the electron transport chain (ETC) proteins (Fig 2D). Interestingly, complex V, ATP Synthase, was not altered. Levels of transcript encoding these OXPHOS subunits were not significantly decreased (Fig 2E), signifying that a post-transcriptional mechanism facilitates the mitochondrial phenotype. We did not observe a decrease in VDAC levels (Fig 2D), suggesting that the ETC reductions do not stem from a broader impairment in mitochondrial biogenesis.

### Thermoneutral housing alleviates BAT inflammation but not depot expansion

**Fig 3:**
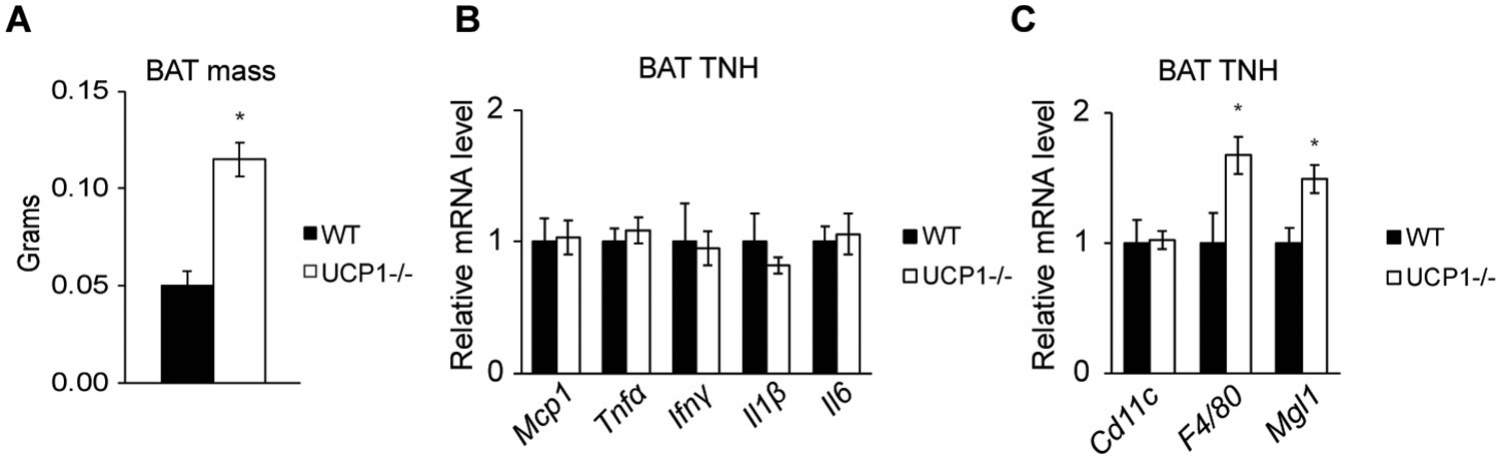
UCP1-/- mice do not exhibit increased markers of inflammation after thermoneutral housing compared to WT mice. Chow-fed, male mice were housed at 30°C for four weeks and sacrificed at 12 weeks of age (n = 5-7/group). (A) BAT mass from WT and UCP1-/- mice. qRT-PCR analysis of markers of macrophage (B) and cytokine and chemokine (C) content in BAT. Data are mean ± SEM. *p<0.05 vs WT.

Since chronic cold stress has been shown to play a critical role in the phenotype of UCP1-/- mice [19, 21], we housed animals at thermoneutrality (30°C) to determine if alleviation of cold stress could abrogate the difference between UCP1-/- and WT mice. Notably, BAT mass was still significantly elevated after four weeks of 30°C housing in UCP1-/- mice compared to thermoneutral housed WT mice (Fig 3A). In contrast, thermoneutral housing completely restored expression of *Mcp1*, *Ifnγ*, *Tnfα*, *Il-1β*, and *Il-6* in brown fat in UCP1-/- mice compared to WT mice (Fig 3B). Gene expression analyses also indicate that elevated infiltration of pro-inflammatory macrophages is lost with thermoneutral housing; *Cd11c* mRNA abundance is restored to WT levels and the 9-fold increase in *F4/80* is reduced to 1.6-fold increase (Fig 3C).

### Thermoneutral housing abrogates BAT UPR in UCP1-/- mice

**Fig 4:**
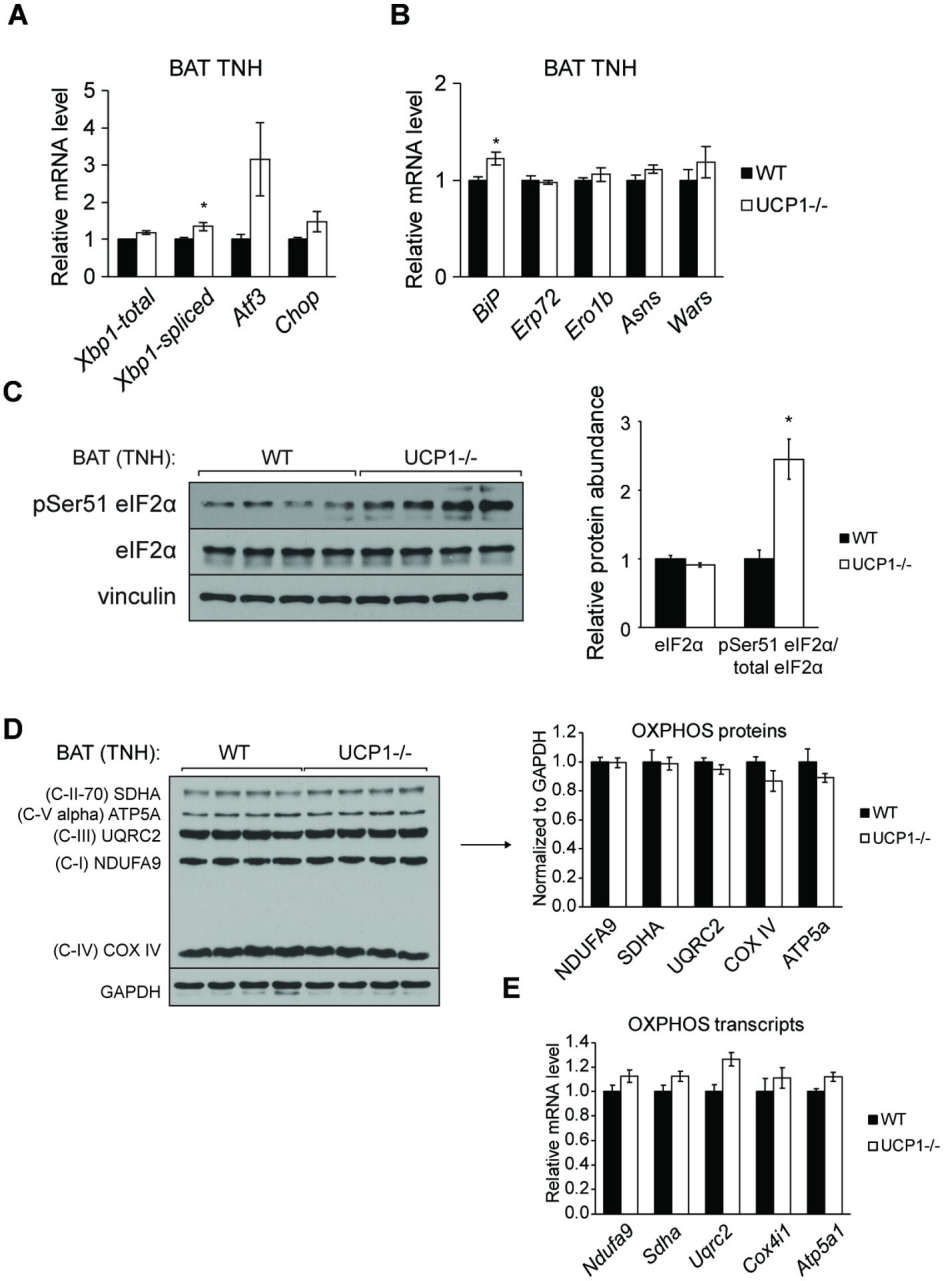
Thermoneutral housing attenuates BAT ER stress in UCP1-/- mice. Chow-fed, male mice were housed at 30°C for four weeks and sacrificed at 12 weeks of age (n = 6-8/group). qRT-PCR analysis of transcription factors (A) and chaperones and amino acid metabolism (B) genes associated with the unfolded protein response. (C) Immunoblotting of eIF2α phosphorylation at Ser51 in WT and UCP1-/- brown adipose tissue. OXPHOS protein (D) and transcript (E) levels of BAT from WT and UCP1-/- mice housed at thermoneutrality. Data are mean ± SEM. *p<0.05 vs WT.

**Fig 5:**
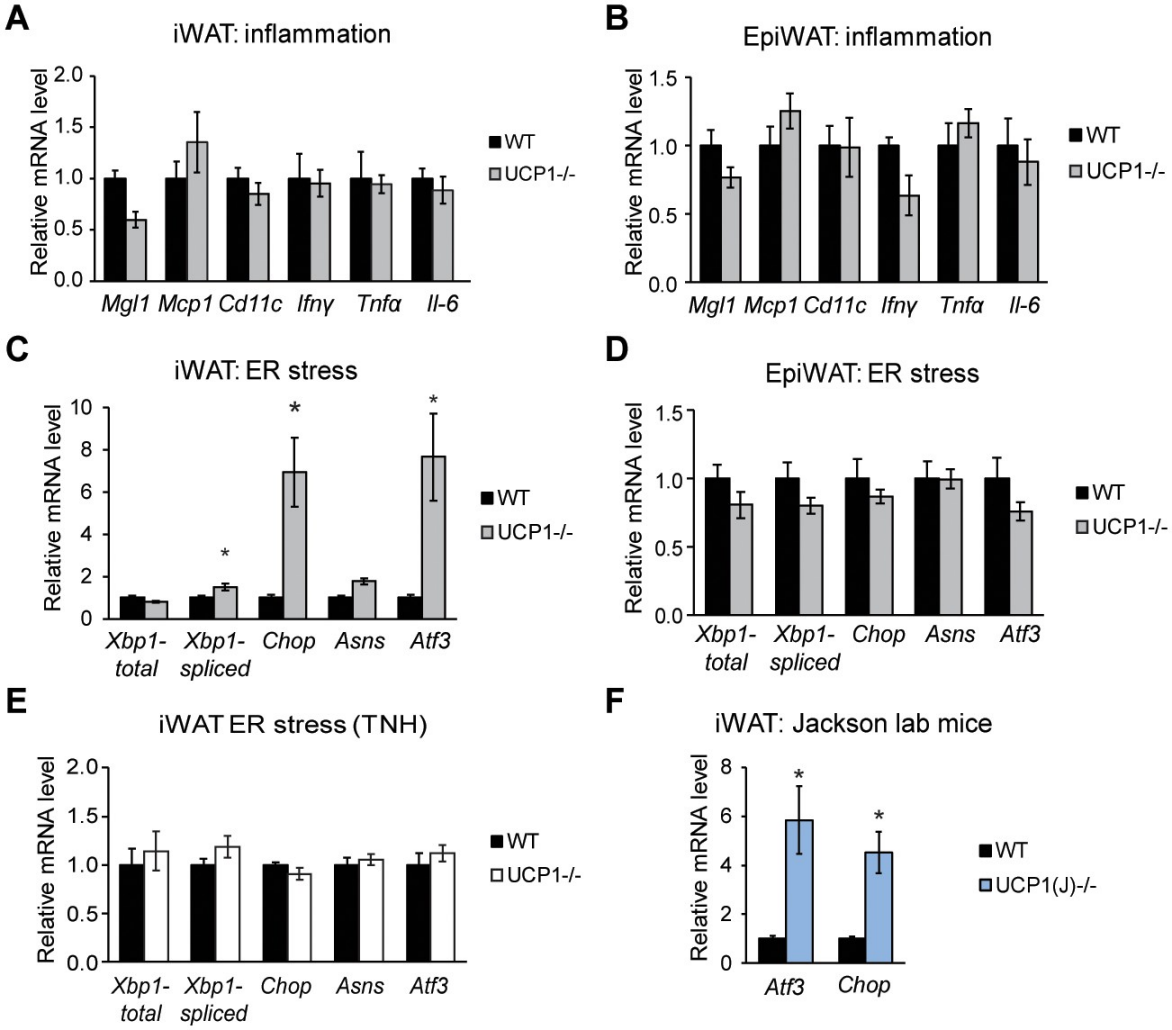
UCP1-/- mice display modestly increased inguinal WAT ER stress gene expression but unaltered expression of inflammation genes. Transcript levels of pro-inflammatory genes in inguinal (A) and epididymal (B) WAT (n = 6/group). Transcript levels of genes associated with ER stress in inguinal (C) and epididymal (D) WAT (n = 6/group). (E) Inguinal WAT ER stress gene expression from WT and UCP1-/- animals housed at thermoneutrality for four weeks (n = 5-7/group). (F) *Atf3* and *Chop* transcript abundance in inguinal WAT from WT and UCP1-/- mice from Jackson labs (UCP1(J)-/-) (n = 5-6/group). Data are mean ± SEM. *p<0.05 vs WT.

Expression of BAT ER stress gene markers was also largely restored to WT levels (Fig 4A and 4B). Notably, *Atf3* and *Chop* were not significantly increased in UCP1-/- mice compared to WT mice after thermoneutral housing (Fig 4A). UCP1-/- mice exhibited a 2.5-fold increase in phosphorylation of eIF2α after thermoneutral housing, but this trend was not as dramatic as observed at room temperature (13-fold increase) (Figs 5C and 2C). Thermoneutral housing also rescued BAT mitochondrial OXPHOS remodeling; electron transport chain protein and transcript levels were not different between UCP1-/- and WT mice housed at thermoneutrality (Fig 4D and 4E). These data show that UCP1 deficiency promotes adipose tissue inflammation and ER stress through chronic cold stress and independently of lipid accumulation and depot expansion.

### Inguinal WAT of UCP1-/- mice exhibits modest ER stress but not pro-inflammation

Since UCP1 deficiency is known to elicit a global phenotype, we surveyed WAT for a potential stress response. Expression of pro-inflammatory genes was not altered in inguinal or epididymal WAT (Fig 5A and 5B). However, we sntraw that inguinal WAT of UCP1-/- mice had modest but significant upregulation of ER stress markers, including spliced *Xbp1*, *Chop* and *Atf3* transcript levels (Fig 5C). Expression of ER stress markers in epididymal WAT was not different between WT and UCP1-/- mice (Fig 5D). To confirm that the ER stress phenotype is not due to the unique nature of our UCP1 ablation, we confirmed elevated *Atf3* and *Chop* gene expression in inguinal WAT of UCP1-/- mice from Jackson labs (the UCP1-/- line most commonly used by the field) (Fig 5F). Since ER stress but not inflammation is present in inguinal WAT of UCP1-/- mice, ER stress and not inflammation is likely to be the instigator of this response.

Collectively, we show that UCP1 deficiency promotes immune cell activation and ER stress in BAT and that UCP1-/- mice do not exhibit greater inflammation and ER stress compared to WT mice after housing at thermoneutrality. Notably, this response is independent of BAT depot expansion.

## Discussion

Mammals are required to adapt to a wide range of environmental conditions like changes in temperature, and UCP1 and brown adipose tissue thermogenesis play a critical role in adaptation to cold. We recently reported the identification of a UCP1 splicing mutation in an SCD3-/- mouse line [19]. Here, we use this UCP1-/- mouse model to reveal the effects of UCP1 deficiency on BAT inflammation and ER stress and determine the temperature-dependency of this phenotype.

First, we demonstrated the development of a pro-inflammation immune state in BAT of UCP1-/- mice. We observed an increase in macrophage markers, pro-inflammatory cytokines, and chemokines, supported by crown-like structures enveloping adipocytes. Synthesis of inflammatory factors by adipocytes, as well as by the infiltrating immune cells, likely also contributes to the inflammatory status. Interestingly, *Mgl1* transcript levels were modestly elevated in UCP1-/- mice with either room temperature- and thermoneutral-housing (Fig 3C), indicating a low, basal presence of anti-inflammatory macrophages that is independent of housing temperature. Additionally, we determined that chronic cold stress drives this response, as UCP1-/- animals housed at 30°C for 4 weeks exhibited minimal to no inflammation or ER stress compared to WT animals housed at 30°C. Our findings are consistent with recently published datasets suggesting an immune response in BAT of UCP1-/- mice [7, 15]. Specifically, transcriptomics conducted by Keipert and colleagues indicated an elevation of immune response-related genes in room temperatures-housed UCP1-/- mice compared to WT mice [15]. Another study utilized quantitative proteomic profiling to identify upregulation of host defense proteins in BAT of cold-exposed UCP1-/- mice and determined that housing animals at thermoneutrality alleviated this response [7].

Next, we identified ER stress as an accompanying comorbidity to pro-inflammatory immune cell infiltration. Phosphorylation of eIF2α and expression of the downstream targets *Atf3* and *Chop* were dramatically upregulated, and *Xbp1* induction and splicing, consequences of ATF6 and IRE1 activation, respectively, were upregulated [22]. We also observed reductions in respiratory chain components. While some groups have reported minimal alterations in oxidative phosphorylation capacity [23] or protein abundance [18] with UCP1 deficiency, our results are consistent with numerous recent studies showing depletion of OXPHOS proteins [7, 24].

Our experiments also shed light on the association between lipid accumulation and adipose tissue UPR activation and inflammation. While adipocyte hypertrophy often correlates with pro-inflammation and the ER stress response [2, 3, 5, 8–10], we determined that the deleterious consequences of UCP1 deficiency on immune and organelle homeostasis are not driven by lipid excess and lipotoxicity. Indeed, our thermoneutral housing experiment dissociates the stress response from the gross BAT depot expansion; UCP1-/- mice did not have BAT inflammation, ER stress, and OXPHOS remodeling but still had elevated BAT depot mass and whitening compared to WT mice after housing at 30°C.

Despite this progress in understanding, several gaps in knowledge remain and it is unclear how chronic cold stress causes this phenotype. A demand for synthesis and folding of the suite of cold-responsive proteins could spur the UPR. Bartelt et. al. reported that cold-induced BAT thermogenesis requires proteasomal activity and proteome remodeling [25]. Alternatively, norepinephrine could directly potentiate this response. Supporting the latter theory, norepinephrine was shown to induce ER stress through activation of PERK in PC12 cells, a neuroendocrine cell model, and β_3_-adrenergic stimulation increased IRE1 activation in brown adipocytes [26, 27]. The relationship between the BAT stress response and reported alterations mitochondrial biology also requires additional attention, as it is also possible that mitochondrial dysfunction contributes to the observed perturbations in ER homeostasis. Kazak et. al. demonstrated that UCP1-deficient BAT mitochondria have reduced calcium-buffering capacity [7], and disruption of ER calcium homeostasis is known to perturb protein chaperone function and activate UPR [28]. Interestingly, cold has been reported to alternatively activate macrophages in adipose tissue [29]; however, we did not observe increases in anti-inflammatory markers.

The directionality between ER stress and inflammation in this context is not clear, as reports indicate that both responses can spur the other [1, 30–32]. Our survey of WAT and preliminary findings that ER stress, but not inflammation (Fig 5A and 5C), manifests in inguinal WAT suggests that inflammation is not necessary to induce the UPR. Nevertheless, additional experiments are required to delineate these trends and determine causality in BAT.

This study has certain limitations. Follow-up experiments in wildtype mice will be needed to determine if this is purely an adaptation to cold or rather an effect of cold only in the context of UCP1 deficiency. Future studies will also be needed to better understand the physiological function of this stress response in BAT and if this phenotype manifests in humans adopting cold exposure as a weight loss method. Indeed, while we have established the impact of UCP1 deficiency on BAT inflammation and ER stress, it remains unclear if the observed BAT stress response impacts other aspects of BAT physiology, such as plasma triglyceride clearance and insulin sensitivity [33, 34].

Collectively, we have unveiled a unique instance of inflammation and ER stress in BAT. Driven by chronic cold stress, and not obesification of a fat depot, loss of UCP1 promotes immune cell infiltration and UPR activation. These findings indicate that the effects of UCP1 deficiency extend beyond impaired brown adipocyte mitochondrial uncoupling and demonstrate that loss of UCP1 also impacts ER homeostasis and elicits an inflammatory response in BAT.

## Supporting information

S1 Table Primer sequences used for qPCR. (XLSX)
